# Safety and efficacy of the tumor-selective adenovirus enadenotucirev with or without paclitaxel in platinum-resistant ovarian cancer: a phase 1 clinical trial

**DOI:** 10.1136/jitc-2021-003645

**Published:** 2021-12-10

**Authors:** Victor Moreno, Maria-Pilar Barretina-Ginesta, Jesús García-Donas, Gordon C Jayson, Patricia Roxburgh, Raúl Márquez Vázquez, Agnieszka Michael, Antonio Antón-Torres, Richard Brown, David Krige, Brian Champion, Iain McNeish

**Affiliations:** 1 START Madrid-FJD, Hospital Universitario Fundación Jiménez Díaz, Madrid, Spain; 2 Medical Oncology, Catalan Institute of Oncology, Girona, Spain; 3 Girona Biomedical Research Institute (IDIBGI), Department of Medical Sciences, University of Girona, Girona, Spain; 4 Medical Oncology, HM Hospitales Centro Integral Oncologico Clara Campal, Madrid, Spain; 5 Department of Medical Onclogy, The Christie Hospital NHS Trust, Manchester, UK; 6 Division of Cancer Sciences, The University of Manchester, Manchester, UK; 7 Institute of Cancer Sciences, Wolfson Wohl Cancer Research Centre, University of Glasgow, Glasgow, UK; 8 Medical Oncology, Beatson West of Scotland Cancer Centre, Glasgow, UK; 9 Medical Oncology, Gynecologic Oncology Unit, MD Anderson Cancer Center Madrid, Madrid, Spain; 10 Medical Oncology, Royal Surrey County Hospital, Guildford, UK; 11 Medical Oncology, Hospital Universitario Miguel Servet, Zaragoza, Spain; 12 PsiOxus Therapeutics Ltd, Abingdon, UK; 13 Ovarian Cancer Action Research Centre, Department of Surgery and Cancer, Imperial College London, London, UK

**Keywords:** immunotherapy, clinical trials as topic, oncolytic viruses, therapies, investigational

## Abstract

**Background:**

Treatment outcomes remain poor in recurrent platinum-resistant ovarian cancer. Enadenotucirev, a tumor-selective and blood stable adenoviral vector, has demonstrated a manageable safety profile in phase 1 studies in epithelial solid tumors.

**Methods:**

We conducted a multicenter, open-label, phase 1 dose-escalation and dose-expansion study (OCTAVE) to assess enadenotucirev plus paclitaxel in patients with platinum-resistant epithelial ovarian cancer. During phase 1a, the maximum tolerated dose of intraperitoneally administered enadenotucirev monotherapy (three doses; days 1, 8 and 15) was assessed using a 3+3 dose-escalation model. Phase 1b included a dose-escalation and an intravenous dosing dose-expansion phase assessing enadenotucirev plus paclitaxel. For phase 1a/b, the primary objective was to determine the maximum tolerated dose of enadenotucirev (with paclitaxel in phase 1b). In the dose-expansion phase, the primary endpoint was progression-free survival (PFS). Additional endpoints included response rate and T-cell infiltration.

**Results:**

Overall, 38 heavily pretreated patients were enrolled and treated. No dose-limiting toxicities were observed at any doses. However, frequent catheter complications led to the discontinuation of intraperitoneal dosing during phase 1b. Intravenous enadenotucirev (1×10^12^ viral particles; days 1, 3 and 5 every 28-days for two cycles) plus paclitaxel (80 mg/m^2^; days 9, 16 and 23 of each cycle) was thus selected for dose-expansion. Overall, 24/38 (63%) patients experienced at least 1 Grade ≥3 treatment-emergent adverse event (TEAE); most frequently neutropenia (21%). Six patients discontinued treatment due to TEAEs, including one patient due to a grade 2 treatment-emergent serious AE of catheter site infection (intraperitoneal enadenotucirev monotherapy). Among the 20 patients who received intravenous enadenotucirev plus paclitaxel, 4-month PFS rate was 64% (median 6.2 months), objective response rate was 10%, 35% of patients achieved stable disease and 65% of patients had a reduction in target lesion burden at ≥1 time point. Five out of six patients with matched pre-treatment and post-treatment biopsies treated with intravenous enadenotucirev plus paclitaxel had increased (mean 3.1-fold) infiltration of CD8 +T cells in post-treatment biopsies.

**Conclusions:**

Intravenously dosed enadenotucirev plus paclitaxel demonstrated manageable tolerability, an encouraging median PFS and increased tumor immune-cell infiltration in platinum-resistant ovarian cancer.

**Trial registration number:**

NCT02028117.

## Introduction

Ovarian cancer is the eighth most common cause of cancer-related death in women, resulting in approximately 185,000 deaths worldwide in 2018.^1^ Most patients are diagnosed with disseminated intraperitoneal (IP) disease.^2^ Although a number of novel therapies are now available, improving outcomes for patients with recurrent platinum-refractory/resistant disease remains extremely challenging.^2^ To date, immuno-oncology approaches have also demonstrated limited success in ovarian cancer,^3^ and novel therapeutic strategies are urgently needed. IP delivery of chemotherapy has been assessed in newly diagnosed ovarian cancer, demonstrating improved survival outcomes, but with increased toxicity, partially related to IP catheter complications.^4 5^

Enadenotucirev is a group B Ad11p/Ad3 chimeric adenoviral vector that was generated by directed evolution to have potent tumor-selective cytotoxicity.^6^ Enadenotucirev is blood stable^7^ and selectively replicates in cells derived from epithelial tumors, leading to local amplification and specific killing of malignant cells by a rapid non-apoptotic immunogenic mechanism.^6 8^

Phase 1 studies of enadenotucirev in epithelial malignancies have demonstrated that both intravenous and intratumoral dosing of enadenotucirev leads to selective delivery to tumor cells and viral persistence, as measured by nuclear hexon staining and detection of viral genomic DNA using quantitative PCR (qPCR) in tumor samples up to ~7 weeks after dosing.^9^ Additionally, enadenotucirev appears to stimulate immune-cell infiltration within cancer cell nests.^9^ A further phase 1 study (EVOLVE, NCT02028442) in epithelial tumors demonstrated a predictable and manageable safety profile with intravenous doses of enadenotucirev, with a maximum tolerated dose (MTD) determined as 3×10^12^ viral particles (vp) when given as repeating dosing cycles every 1 or 3 weeks.^10^ The most commonly reported grade ≥3 treatment-emergent adverse events (TEAEs) were hypoxia, lymphopenia, and neutropenia. A dose-independent alpha half-life of 16.7 min was observed with intravenous dosing, consistent with rapid clearance of the virus by hepatic Kupffer cells. Consistent with published data for the Ad11 serotype (identical to the enadenotucirev capsid),^11^ pre-existing antibody immunity to enadenotucirev was low or absent. However, following enadenotucirev administration, all patients displayed an antibody response, with the increase typically plateauing by day 20 and being sustained thereafter; this response was not boosted further by repeated dosing cycles. Transient dose-dependent cytokine increases (including interferon (IFN)-γ, interleukin-6, C-C motif chemokine ligand 2 and tumor necrosis factor alph) were observed following enadenotucirev dosing, with increases greater following the first dose than after any subsequent doses. The EVOLVE study was not designed to assess efficacy, but stable disease for >12 weeks was observed for five patients.^10^

The significant unmet need in platinum-resistant ovarian cancer, and the potential to assess pharmacodynamic activity in peritoneal biopsies, made ovarian cancer an attractive target for assessing a novel tumor-selective virus. Additionally, the potential to use IP delivery of enadenotucirev in ovarian cancer could avoid rapid clearance of vp by the liver and lead to longer viral persistence in the peritoneal cavity. Previous preclinical assessments have also demonstrated potential synergy between oncolytic adenoviruses and microtubule disrupting drugs.^12^ Therefore, we assessed the activity of enadenotucirev in combination with paclitaxel using a murine model of ovarian cancer before conducting a phase 1 dose-escalation and dose-expansion study in platinum-resistant epithelial ovarian cancer.

## Methods

### Preclinical analysis of enadenotucirev activity

An in vivo murine model of ovarian cancer was developed by implanting female CB17 severe combined immunodeficiency mice with luciferase-expressing SKOV-3 (human ovarian carcinoma) cells via IP injection (2.5×10^6^ cells per mouse) and subsequently dosing with paclitaxel, enadenotucirev, both combined or vehicle (phosphate-buffered saline; PBS) via IP injection. Disease progression was monitored using an in vivo imaging system, as previously described.^13^

All mice were housed and treated in accordance with UK Home Office guidelines outlined in the Animals (Scientific Procedures) Act 1986.

### Study design

**Figure 1:**
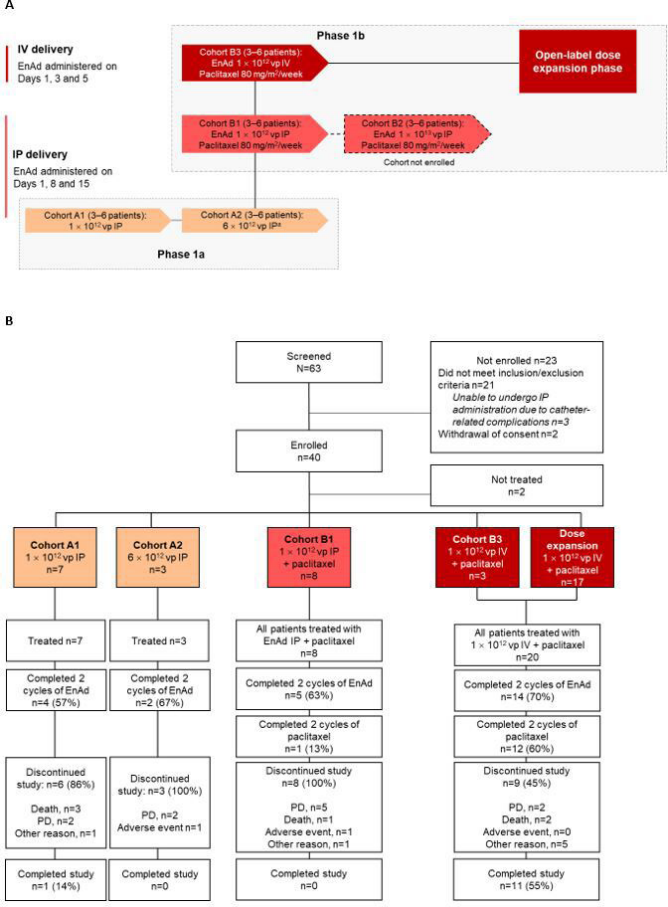
OCTAVE study design (A) and patient disposition (B). (A) IP enadenotucirev monotherapy escalation (phase 1a) was conducted in parallel with the phase 1a combination dose-escalation, starting when the first feasible level of enadenotucirev monotherapy was determined in Phase Ia. In the combination therapy cohorts, paclitaxel was given on days 9, 16 and 23 of each cycle. (B) OCTAVE study patient disposition. ^a^Planned dose level of 1×10^13^. EnAd, enadenotucirev; IP, intraperitoneal; IV, intravenous; PD, progressive disease; vp, viral particle.

The multicenter open-label non-randomized OCTAVE study (ColoAd1-2001; NCT02028117) included phase 1a and 1b dose-escalation and dose-expansion stages (figure 1A). Phase 1a was a standard 3+3 dose-escalation stage, with patients receiving two 4-week cycles of enadenotucirev monotherapy delivered by IP injection. A phase 1b dose-finding stage then assessed the dose of enadenotucirev to be used in combination with intravenous paclitaxel. Once an enadenotucirev dose regimen was selected in combination with paclitaxel, patients were then enrolled in an open-label dose-expansion phase to assess efficacy and further characterize safety. OCTAVE was initially designed to assess IP dosing of enadenotucirev, but (as described later) an intravenous dosing arm was subsequently added to the study based on emerging data from the IP cohorts and other studies of intravenous administered enadenotucirev.^9 10^

### Participants

Eligible patients had histologically confirmed non-resectable epithelial ovarian, fallopian tube or primary peritoneal cancer. Patients were required to have to platinum-resistant disease (defined as disease progression within 6 months of receiving prior platinum-based chemotherapy), or—in phase 1a and for the first three patients enrolled in phase 1b only—to have no standard-of-care treatment options. Additional eligibility criteria included: age ≥18 years, Eastern Oncology Cooperative Group performance status of 0–1, and adequate renal, hepatic, bone marrow, and coagulation function. Key exclusion criteria included: tumors of malignant mixed mesodermal/carcinosarcoma or mucinous subtypes, or non‐epithelial ovarian cancers; history or evidence of significant immunodeficiency, renal or autoimmune disease; or recent use of antiviral agents (ribavirin, adefovir, lamivudine or cidofovir within 7 days prior to day 1; pegylated IFN withing 14 days prior to day 1). Notably, patients who had received prior weekly paclitaxel for platinum-resistant disease were eligible for the OCTAVE study.

### Procedures

In phase 1a, dose-escalation patients were treated with two cycles of IP administered enadenotucirev monotherapy on days 1, 8 and 15 of each 28-day cycle (cohorts A1 and A2, figure 1A). An initial starting dose of 1×10^12^ vp was chosen based on preclinical findings in murine models, and had been previously tested as an intravenous dose in another phase 1 study.^10^ Dose-escalation was conducted up to a highest planned dose level of 1×10^13^ vp on days 1, 8 and 15 of each 28-day cycle. Dose-escalation decisions were based on the 3+3 model, with tolerability assessed in the first three evaluable patients treated at each dose level, and a further three patients enrolled if one dose limiting toxicity (DLT) was observed. In this model, the MTD was the highest dose level associated with a DLT rate of <17% (DLTs in zero out of three or one out of six patients).

In phase 1b, patients received two 28-day cycles of treatment, with enadenotucirev given either IP on days 1, 8 and 15 of each cycle (cohorts B1 and B2, figure 1A) or intravenously on days 1, 3 and 5 of each cycle (cohort B3, figure 1A). In all cohorts in phase 1b, paclitaxel was given at a fixed intravenous dose of 80 mg/m^2^ on days 9, 16 and 23 of each cycle to allow time for the biological effect of enadenotucirev to be established. Dose-escalation of enadenotucirev (intravenous or IP) plus paclitaxel was planned, starting at the lowest feasible dose identified in phase 1a and using the same 3+3 model, to determine the dose of enadenotucirev recommended in combination with weekly intravenous paclitaxel. During the dose-expansion phase enadenotucirev and paclitaxel were given at the dose selected in phase 1b dose-escalation.

#### Safety and tolerability

The incidence, nature, and severity of AEs were characterized using the National Cancer Institute Common Terminology Criteria for Adverse Events V.4.03. In phase 1a and phase 1b dose-escalation, a clinical event committee (CEC) composed of one independent oncologist, a representative of the Sponsor with a medical background and all Investigators participating in the phase 1 part of the study reviewed all safety data (including any DLTs). The CEC was responsible for expanding a dose level cohort, authorizing enrolment at the next dose level, determining subsequent dose levels and potential changes to the administration schedules, stopping the dose-escalation and determining the dose recommended for phase 1b and the dose-expansion phase. During the 28-day DLT period (from first administration of enadenotucirev), any of the following toxicities were defined as DLTs if they were considered at least possibly attributed to enadenotucirev, whether given as monotherapy or in combination with paclitaxel:

- Grade ≥3 non-hematological AEs lasting >3 days despite optimal supportive care, with the exception of alopecia or self-limiting or medically controllable toxicities (eg, nausea, vomiting, diarrhea, fatigue, headache, chills, electrolyte disturbances, hypersensitivity reactions).
- Febrile neutropenia, when the neutropenia was considered at least possibly due to treatment with enadenotucirev.
- Grade 4 neutropenia or thrombocytopenia lasting for more than 14 days.
- Any AE resulting in fewer than three doses of enadenotucirev being administered within 28 days from first administration of enadenotucirev in phases 1a or 1b or fewer than three doses of paclitaxel being administered over 28 days from first administration of enadenotucirev in phase 1b.

#### Efficacy assessments

In phase 1b and the dose-expansion phase, tumor imaging (computed tomography (CT) with oral and intravenous contrast) was performed prior to treatment, as clinically indicated, and at 8-week intervals during the study. A second scan after ≥4 weeks was required to confirm responses or disease progression.

#### Histopathology and immunohistochemistry

Tumor biopsies were collected from patients from the dose-expansion phase and patients from phase 1b treated at the dose recommended for dose-expansion phase. Biopsies were taken at baseline and between days 33 and 37 (~5 weeks after first enadenotucirev dosing). Sections were stained for CD8, Granzyme B, Ki67, CD4, programmed death-1 (PD-1) and programmed death-ligand 1 (PD-L1) (online supplemental appendix).

10.1136/jitc-2021-003645.supp1 Supplementary data

#### Gene expression profiling

Gene expression analysis was conducted using formalin-fixed paraffin embedded tumor tissue from pre-treatment and post-treatment biopsies using the nCounter Analysis System (Nanostring Technologies) with the PanCancer Immune Profiling Panel codeset and a custom-designed codeset of 30 genes as described in online supplemental appendix.

#### Detection of enadenotucirev in tumor samples by qPCR

Detection of enadenotucirev in frozen tumor tissue from pre-treatment and post-treatment biopsies was performed by qPCR of the E3 gene as described in the online supplemental appendix.

#### Pharmacokinetics and pharmacodynamics

Pharmacokinetic samples (blood for all patients and peritoneal fluid for patients receiving IP treatment) were taken pre-dose and post-dose during phase 1a (immediately prior to treatment, and at 1 hour (blood sample only), 3 hours and 9 hours after end the end of administration). The concentration of enadenotucirev in the blood was measured using qPCR to detect genomic viral DNA.^9^

Enadenotucirev shedding was assessed during dose-escalation only and required rectal and buccal swabs, as well as urine samples for measurement of genome copies by qPCR.^9^ Serum anti-enadenotucirev antibody response was assessed using a Meso Scale Discovery (Meso Scale Diagnostics, Rockville, Maryland, USA) ELISA by BioOutsource (Glasgow, UK).^9^ For patients in the IP cohort, two 5 mL serum samples and two 5 mL peritoneal samples were taken at days 1, 29 and 50/51 and at the end of study treatment visit 28 days (±3 days) after the last administration of enadenotucirev (peritoneal fluid only if the peritoneal catheter was still in place). For patients in the intravenous cohort, two 5 mL serum samples were taken on days 1, 29 and 51.

### Objectives and endpoints

For phase 1a and phase 1b, the primary objective was to determine the MTD of enadenotucirev (with paclitaxel in phase 1b). In the dose-expansion phase, the primary endpoint was progression-free survival (PFS) with intravenous enadenotucirev plus paclitaxel, as assessed by an independent central reviewer per RECIST V.1.1.^14^

Secondary endpoints in all phases included safety and tolerability; overall survival (OS); objective response rate (ORR), duration of response (DoR) and clinical benefit rate per RECIST V.1.1, immune-related response criteria (irRC)^15^ and GCIG CA-125 criteria^16^; PFS; and immune responses to enadenotucirev. Additional secondary endpoints in phase 1 only were viral kinetics and shedding.

### Statistical analysis

No formal sample size calculations were performed for phase 1a/b. For dose-expansion, a planned sample size of 20 evaluable patients was selected to provide 80% power to detect an improvement in PFS of 20% (based on a historical 16-week PFS rate of 55%^17^ and a one-sided significance level of 0.15). As 20%–25% of patients were expected to be non-evaluable, patients in phase 1b treated with the dose regimen used in the dose-expansion stage were pooled with patients from the dose-expansion phase with the aim of achieving a target sample size of 26 patients.

The safety analysis set included all patients who received ≥1 dose of study treatment. Response rates, PFS and OS were assessed using the full analysis set, which included all patients with ≥1 dose of study treatment, as well as a baseline and at least one post-baseline efficacy assessment.

Time-to-event endpoints were calculated using Kaplan-Meier estimates and associated two-sided 95% CIs were generated using log-log transformation.

PFS was defined as time from first dose to disease progression or death, whichever occurred first. Patients whose disease did not progress and who did not die before the end of the study, who had withdrawn before the end of study, died more than 4 months after the last clinical/radiological assessment or did not have a post-baseline disease assessment were censored at the date of last evaluable clinical/radiological assessment.

Response rates were descriptively summarized with corresponding two-sided 95% CIs based on Clopper-Pearson method.

## Results

### Preclinical evaluation of enadenotucirev combination therapy in a murine model of ovarian cancer

IP injection of enadenotucirev or paclitaxel reduced tumor burden versus negative control. Combining enadenotucirev and paclitaxel resulted in a significantly greater reduction in tumor burden than paclitaxel alone, and combination with paclitaxel did not reduce the efficacy of enadenotucirev (online supplemental figure 1).

### Patient characteristics and disposition

Across all cohorts of the phase 1 study, a total of 63 patients were screened between June 2014 and March 2019 and 38 patients were treated at three sites in the UK and five sites in Spain (figure 1). All treated patients were included in both the safety and full analysis sets. Baseline demographics were similar across cohorts (table 1), with all patients having metastatic platinum-resistant disease at enrolment. All patients had received paclitaxel and carboplatin prior to enrolment, with the majority (76%) receiving multiple lines of paclitaxel-containing therapy (two prior lines, 66%; three prior lines, 11%) prior to enrolling. In total, 24% of patients received paclitaxel monotherapy after failure of prior paclitaxel in combination with carboplatin.

**Table 1:** Baseline demographics

	Enadenotucirev monotherapy		Enadenotucirev plus paclitaxel		All patients (N=38)
Characteristic	1×10^12^ IP (n=7)	6×10^12^ IP (n=3)	1×10^12^ IP (n=8)	1×10^12^ IV (n=20)	
Median age, years (min, max)	68 (54, 77)	64 (47, 68)	60 (53, 70)	59 (36, 76)	63 (36, 77)
ECOG performance status					
0–1	6 (86)	3 (100)	7 (88)	20 (100)	36 (95)
2	0	0	1 (13)*	0	1 (3)
Missing	1 (14)	0	0	0	1 (3)
Median time from diagnosis to screening, months (min, max)	42.8 (13.7, 90.9)	28.0 (14.9, 50.0)	50.6 (33.9, 109.7)	39.0 (7.2, 278.7)	41.8 (7.2, 278.7)
Histological type					
Serous adenocarcinoma	4 (57)	3 (100)	5 (63)	15 (75)	27 (71)
Endometrioid adenocarcinoma	1 (14)	0	0	1 (5)	2 (5)
Other	2 (29)	0	3 (38)	4 (20)	9 (24)
Median (min, max) CA-125 (U/mL)	8750 (93, 29520)	114 (108, 120)	362 (135, 12000)	138 (30, 3958)	151 (30, 29520)
n	3	2	7	17	29
Median (min, max) prior regimens	6 (3, 6)	4 (2, 6)	6 (4, 8)	4 (1, 12)	5 (1, 12)
Prior chemotherapy	7 (100)	3 (100)	8 (100)	20 (100)	38 (100)
Prior paclitaxel	7 (100)	3 (100)	8 (100)	20 (100)	38 (100)
Prior hormonal therapy	2 (29)	1 (33)	2 (25)	4 (20)	9 (24)
Prior monoclonal antibodies	1 (14)	1 (33)	6 (75)†	13 (65)	21 (55)
Prior bevacizumab	1 (14)	1 (33)	6 (75)	12 (60)	20 (53)
Prior ipilimumab	0	0	1 (13)	0	1 (3)
Prior nivolumab	0	0	1 (13)	0	1 (3)
Other	0	0	0	1 (5)	1 (3)
Prior cancer-related surgery	7 (100)	3 (100)	8 (100)	17 (85)	35 (92)
Interval from last taxane-based chemotherapy					
<6 months	1 (14)	3 (100)	0	4 (20)	8 (21)
≥6 months	6 (86)	0	8 (100)	16 (80)	30 (79)

Data are n (%) unless specified otherwise.

*One patient had and ECOG performance status of 1 at screening and two at baseline.

†One patient received prior bevacizumab, ipilimumab, and nivolumab.

ECOG, Eastern Oncology Cooperative Group; IP, intraperitoneal; IV, intravenous.

At the data cut-off (November 29, 2019), median (min–max) follow-up in the dose-expansion cohort was 7.4 (0.4–14.1) months; median follow-up ranged from 2.3 to 5.7 months in the enadenotucirev IP cohorts. In total, 12/38 (32%) patients completed the study, the majority (11/12) of whom were enrolled in the dose-expansion phase (figure 1B). The most common reasons for discontinuing the study were progression of disease (n=12) and death (n=6).

### Dose-escalation and DLTs

In the phase 1a monotherapy dose-escalation, seven patients received enadenotucirev 1×10^12^ vp IP monotherapy in cohort 1A (figure 1). No DLTs occurred in this cohort and so the next three patients were enrolled at the next dose level (cohort A2). Patients in cohort A2 received a dose of 6×10^12^ vp, with no DLTs observed. Although the maximum planned dose of enadenotucirev in phase 1a was 1×10^13^ vp, this dose level was not assessed due to the occurrence of DLTs when enadenotucirev was delivered intravenously at a dose of 1×10^13^ vp in a separate study in advanced solid tumors (EVOLVE^10^). Although this was via a different route of administration, with a different safety profile to the IP delivery, it was considered prudent not to reassess this dose and end phase 1a dose-escalation.

In the phase 1b combination dose-escalation, eight patients were treated with enadenotucirev at a dose of 1×10^12^ vp IP plus paclitaxel (cohort B1). No protocol-defined DLTs occurred at this dose level; however, the high level of catheter complications related to IP dosing in this cohort led the Investigators to change the design of the study and the IP arm was discontinued. The same dose of enadenotucirev 1×10^12^ vp was therefore instead investigated via intravenous infusion in combination with paclitaxel (cohort B3).

No DLTs were observed in the three patients enrolled in cohort B3. However, based on a review of available safety data from the EVOLVE study,^10^ the CEC decided against further escalation of the dose due to the increased risk of transient neutropenia observed in the EVOLVE study, particularly as neutropenia is also a known side effect for paclitaxel. The enadenotucirev 1×10^12^ vp intravenous dose was, therefore, selected for dose-expansion in combination with paclitaxel. An additional 17 patients were treated in the dose-expansion cohort and were pooled with the three patients enrolled in cohort B3, for a total of 20 patients eligible for efficacy analyses.

### Safety and tolerability

All patients experienced at least one TEAE and 24/38 (63%) patients experienced at least one Grade ≥3 TEAE. The most frequently reported grade≥3 TEAEs was neutropenia/neutrophil count decreased (8 patients (21%)) (table 2). In total, 11 patients (29%) experienced at least 1 grade ≥3 TEAE considered related to enadenotucirev, most commonly neutropenia (n=7) and anemia (n=2); all other events were reported for a single patient only.

**Table 2:** Treatment-emergent adverse events occurring in ≥10% of patients overall

Event, n (%) patients	Enadenotucirev IP monotherapy (n=10)		Enadenotucirev IP plus paclitaxel (n=8)		Enadenotucirev IV plus paclitaxel (n=20)		Overall (N=38)	
	Any grade	Grade ≥3	Any grade	Grade ≥3	Any grade	Grade ≥3	Any grade	Grade ≥3
Gastrointestinal disorders	10 (100)	4 (40)	8 (100)	2 (25)	12 (60)	2 (10)	30 (79)	8 (21)
Diarrhea	7 (70)	3 (30)	4 (50)	0	5 (25)	0	16 (42)	3 (8)
Nausea	7 (70)	0	2 (25)	0	7 (35)	1 (5)	16 (42)	1 (3)
Abdominal pain	5 (50)	0	4 (50)	1 (13)	3 (15)	0	12 (32)	1 (3)
Vomiting	5 (50)	1 (10)	2 (25)	0	3 (15)	1 (5)	10 (26)	2 (5)
Constipation	0	0	2 (25)	0	4 (20)	0	6 (16)	0
Ascites	1 (10)	0	2 (25)	1 (13)	1 (5)	0	4 (11)	1 (3)
General disorders and administration site conditions	8 (80)	0	6 (75)	0	13 (65)	1 (5)	27 (71)	1 (3)
Fever	3 (30)	0	4 (50)	0	4 (20)	0	11 (29)	0
Fatigue	2 (20)	0	2 (25)	0	4 (20)	0	8 (21)	0
Asthenia	1 (10)	0	0	0	6 (30)	0	7 (18)	0
Chills	2 (20)	0	2 (25)	0	3 (15)	0	7 (18)	0
Edema peripheral	2 (20)	0	3 (38)	0	1 (5)	0	6 (16)	0
Influenza-like illness	2 (20)	0	0	0	2 (10)	0	4 (11)	0
Blood/lymphatic system disorders	6 (60)	0	5 (63)	5 (63)	13 (65)	6 (30)	24 (63)	11 (29)
Anemia	6 (60)	0	3 (38)	1 (13)	10 (50)	2 (10)	19 (50)	3 (8)
Neutropenia/neutrophil count decreased	0	0	4 (50)	3 (38)	10 (50)	5 (25)	12 (32)	8 (21)
Infections and infestations	5 (50)	1 (10)	5 (63)	2 (25)	10 (50)	1 (5)	20 (53)	4 (11)
Urinary tract infection	3 (30)	1 (10)	0	0	2 (10)	0	5 (13)	1 (10)
Nervous system disorders	6 (60)	1 (14)	3 (38)	0	6 (30)	0	15 (39)	1 (3)
Lethargy	4 (40)	1 (14)	2 (25)	0	1 (5)	0	7 (18)	1 (3)
Headache	3 (30)	0	1 (13)	0	1 (5)	0	5 (13)	0
Respiratory, thoracic and mediastinal disorders	3 (30)	0	3 (38)	2 (25)	5 (25)	0	11 (29)	2 (5)
Dyspnea	3 (30)	0	1 (13)	0	2 (10)	0	6 (16)	0
Cough	0	0	2 (25)	0	2 (10)	0	4 (11)	0
Metabolism and nutrition disorders	5 (50)	0	2 (25)	0	3 (15)	0	10 (26)	0
Hypomagnesemia	1 (10)	0	1 (13)	0	2 (10)	0	4 (11)	0
Skin and subcutaneous tissue disorders	1 (10)	0	3 (38)	0	5 (25)	0	9 (24)	0
Alopecia	0	0	2 (25)	0	4 (20)	0	6 (16)	0

IP, intraperitoneal; IV, intravenous.

Twelve patients (32%) experienced at least one treatment-emergent serious adverse event (TESAE). The most frequently reported TESAE was abdominal pain (two patients (5%) overall); all other TESAEs were reported for a single patient only. Five TESAEs were considered related to enadenotucirev: grade 4 left ventricular failure (IP monotherapy), grade 3 diarrhea (IP monotherapy), grade 2 viral infection (IP plus paclitaxel), grade 2 neutropenia (IP plus paclitaxel), and grade 3 staphylococcal skin infection (intravenous plus paclitaxel); all five TESAEs resolved.

In total, six patients discontinued treatment due to TEAEs, including one patient who discontinued enadenotucirev IP monotherapy due to a grade 2 TESAE of catheter site infection. Additional TEAEs related to catheter complications included grade 2 catheter site infection and grade 1 catheter site erythema. One additional AE of grade 2 catheter site pain occurred 2 weeks before the first dose of study treatment and did not resolve in the study period.

Two patients died following TEAEs: one patient who was treated with enadenotucirev IP plus paclitaxel died from septic shock considered unrelated to enadenotucirev and one patient treated with intravenous enadenotucirev died following a SAE of general physical health deterioration. Cause of death was reported as disease progression; however, the event of general physical health deterioration was considered to be possibly related to enadenotucirev.

Transient changes in activated partial thromboplastin time, prothrombin international normalized ratio, and D-Dimer labs were observed following treatment, but were not associated with clinical manifestation of bleeding or clotting events. No evidence of enadenotucirev-related hepatic or renal injury was observed in this study.

### Efficacy

#### Progression-free survival

**Figure 2:**
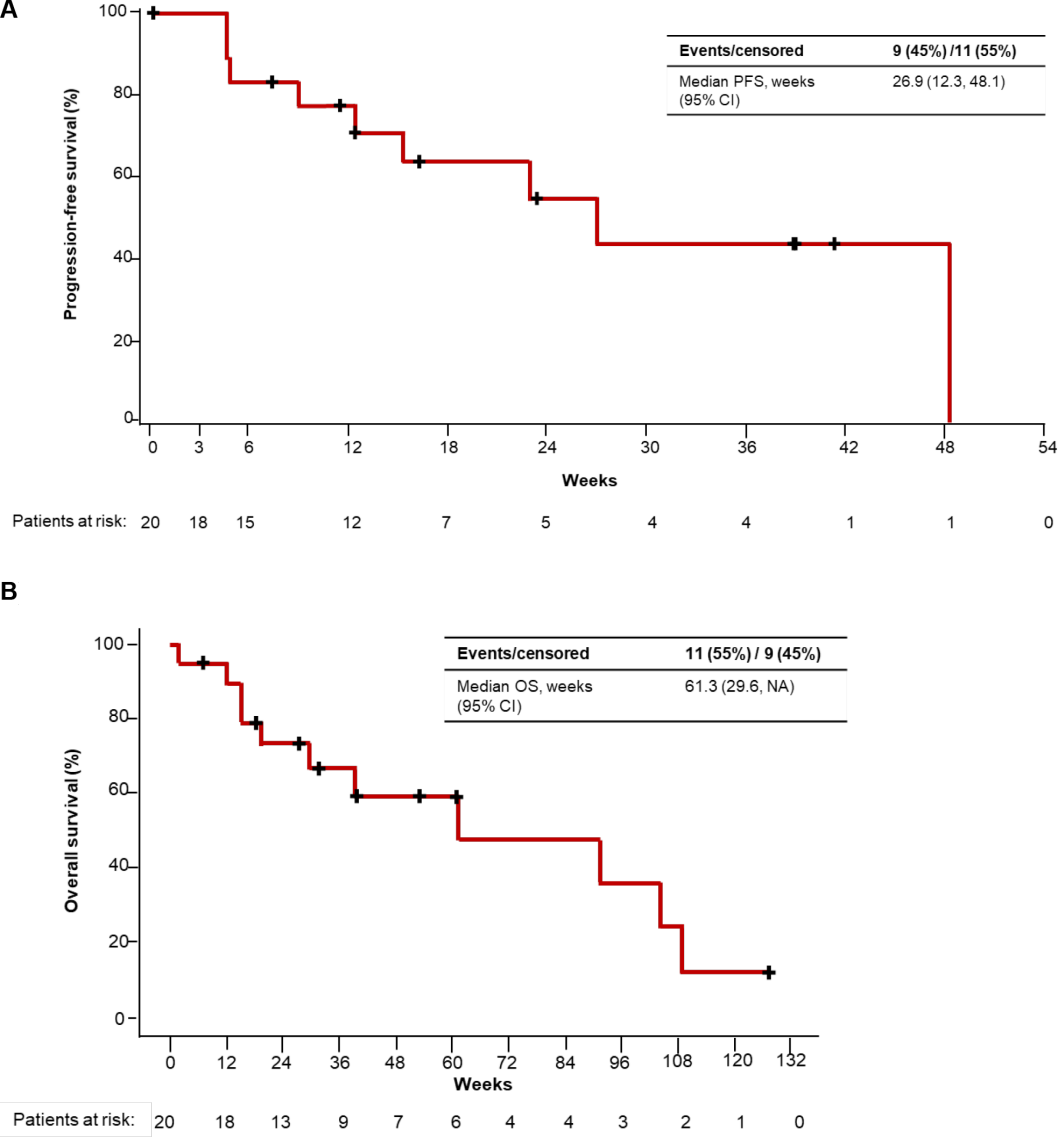
Progression-free survival (PFS) per independent review (A) and long-term follow-up for overall survival (OS) (B) (patients receiving intravenous enadenotucirev plus paclitaxel). NA, not available.

Among the 20 patients who received intravenous enadenotucirev plus paclitaxel, the 4-month PFS rate was 64% (figure 2A). Per Investigator, the 4-month PFS rate was 53% (95% CI 29% to 72%). Median (95% CI) PFS in the intravenous enadenotucirev plus paclitaxel cohorts was 6.2 months (2.8–11.1 months) by Independent assessment and 3.7 months (1.7–5.4 months) by Investigator assessment. PFS according to irRC and GCIG CA-125 criteria was generally similar to that with RECIST V.1.1 (table 3; online supplemental table 1).

**Table 3:** PFS and response rate per Independent assessment (RECIST V.1.1)

	Enadenotucirev IP monotherapy (n=10)	Enadenotucirev IP+paclitaxel (n=8)	Enadenotucirev IV+paclitaxel (n=20)
PFS, % (95% CI)			
Median	1.7 (1.2 to 3.5)	6.4 (1.3 to 6.4)	6.2 (2.8 to 11.1)
4 month PFS rate	11.1 (0.6 to 38.8)	66.7 (19.5 to 90.4)	63.8 (36.1 to 82.1)
6 month PFS rate	0.0 (NE to NE)	66.7 (19.5 to 90.4)	54.7 (26.5 to 76.1)
Best overall response, n (%)			
Complete response	0	0	0
Partial response	0	0	2 (10)
Stable disease	0	2 (25)	7 (35)
Progressive disease	7 (70)	2 (25)	5 (25)
Not evaluable	3 (30)	4 (50)	6 (30)
Overall response rate, %	0	0	10
95% CI	–	–	1 to 32
Clinical benefit rate, %	0	25	45
95% CI	–	3 to 65	23 to 69

IP, intraperitoneal; IV, intravenous; PFS, progression-free survival.

#### Overall survival

At the November 29, 2019 data cut-off, OS remained immature and was not estimatable for patients who received intravenous enadenotucirev plus paclitaxel (online supplemental table 2). Median OS was 8.0 and 6.4 months in the enadenotucirev IP monotherapy and enadenotucirev IP plus paclitaxel cohorts, respectively. An ad hoc extended follow-up analysis for OS for patients treated with intravenous enadenotucirev plus paclitaxel (cut-off: January 11, 2021) demonstrated a median OS of 14.1 months (95% CI 6.8 to NA months) (figure 2B).

#### Response rate

ORR among the 20 patients who received intravenous enadenotucirev plus paclitaxel was 10% per Independent assessment, with two patients achieving partial response (PR). Progressive disease (PD) was reported in 5 (25%) patients, and a further 6 (30%) patients were non-evaluable. No objective responses were observed in the other dose cohorts (table 3). By Investigator assessment, ORR with intravenous enadenotucirev plus paclitaxel was 30% (PR in six patients), with a clinical benefit rate of 40% (online supplemental table 3). Per Investigator assessment, 11 (55%) patients had PD and one (5%) patient was non-evaluable.

**Figure 3:**
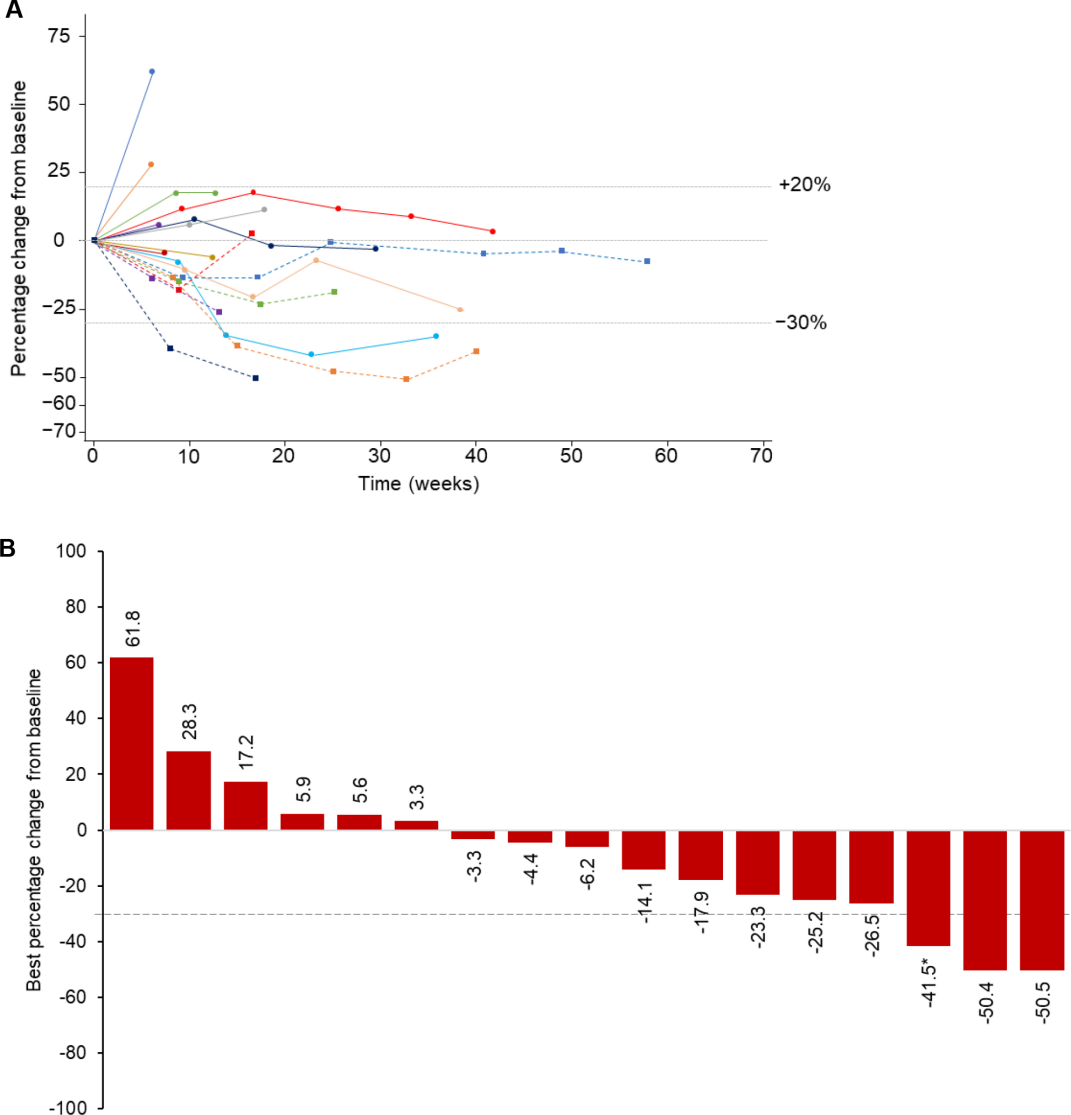
Change in target lesion burden over time (A) and best change in target lesion burden (B) per Independent assessment (RECIST V.1.1; patients receiving intravenous enadenotucirev plus paclitaxel). Evaluable patients: n=17. (A) Percentage change from baseline in target lesion burden over time in the phase 1b intravenous enadenotucirev plus paclitaxel cohort. Each line represents an individual patient. (B) Best percentage change from baseline in target lesion burden (sum of diameters of target lesions per RECIST V.1.1) according to Independent review. Dashed line indicates 30% decrease in target lesion burden. Each bar represents an individual patient. *Patient achieved PR at one assessment, but then had new lesion progression on the confirmatory scan so response was categorized as PD. PD, progressive disease; PR, partial response.

Most patients (65% of evaluable patients per Independent assessment) receiving intravenous enadenotucirev plus paclitaxel had a reduction in target lesion burden at one or more timepoints (figure 3A; online supplemental figure 2A). Per independent review, three patients had a best reduction in target lesion burden ≥30% (figure 3B); however, one patient experienced new lesion progression despite a reduction in target lesion burden and was therefore considered to have a best overall response of PD (PR per Investigator assessment). DoR could not be assessed due to the low number of responders; however, stable reductions in target lesion burden that were maintained for up to 60 weeks were observed (figure 3A). Best reduction in target lesion burden per Investigator review is shown in online supplemental figure 2B).

#### Immune cell infiltration

**Figure 4:**
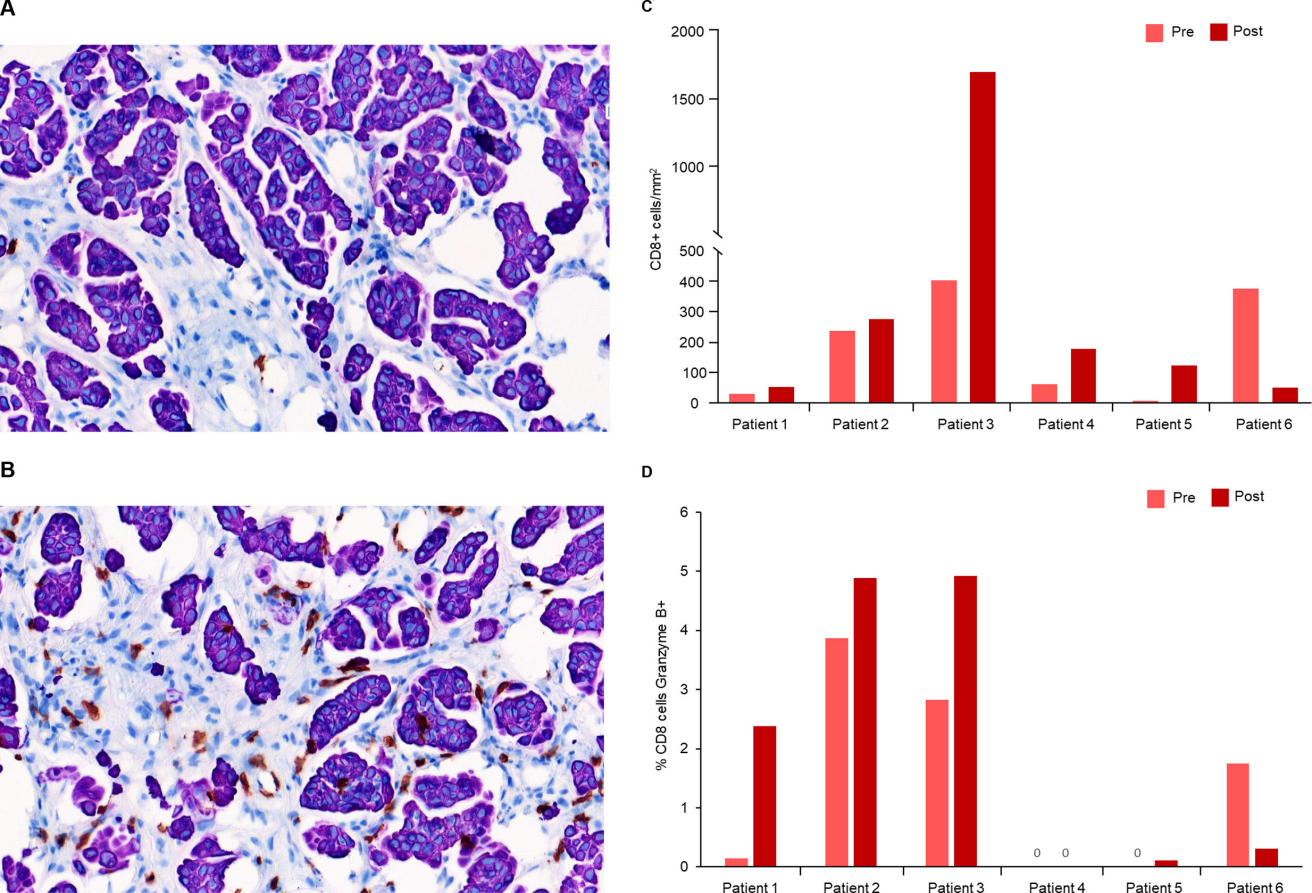
T-cell infiltration and cytotoxic activity after enadenotucirev treatment. Representative pre-treatment (A) and post-treatment (B) biopsy samples after immunohistochemistry (IHC) staining for intratumoral CD8+ T cells (shown in brown). Post-treatment biopsies were taken ~5 weeks after first enadenotucirev dosing. Automated imaging analysis was performed for the six patients treated with enadenotucirev intravenous plus paclitaxel to quantify CD8 +T cell tumor infiltration (C) and percentage of CD8 +cells which were also granzyme B+ (D).

Six patients treated with intravenous enadenotucirev plus paclitaxel had matching pre-treatment and post-treatment tumor biopsy samples with sufficient tumor tissue for immunohistochemistry staining. Cell counting by automated image analysis demonstrated increased intra-tumoral infiltration of CD8+ T-cells in 5/6 patients with post-treatment biopsies (mean 3.1-fold increase, figure 4A–C). An increased proportion of CD8+ T cells that were also granzyme B positive (marker of cytolytic activity) was seen in four in six patients with post-treatment biopsies (mean 4.8-fold increase; figure 4D). With limited patient numbers, no clear relationship between T-cell infiltration or granzyme B activity and OS or response was seen; however, a notable reduction in tumor burden was observed in the patient with the greatest increase in T-cell infiltration (best reduction in target lesion burden per independent review: −25.2%). With limited sample numbers, no clear effect of treatment on CD4+ T-cell infiltration, Ki67 or PD-L1 expression on immune cells or tissue staining for PD-1 was seen in post-treatment biopsies (data not shown).

#### Gene expression profiling

Four paired biopsy samples were available for analysis. While all four paired biopsy samples showed increased expression of some inflammatory/IFN-regulated genes (eg, GZMA, CXCL9) in post-treatment biopsies, the dataset was too small to draw any firm conclusions regarding increased inflammatory gene expression patterns (data not shown).

#### Detection of enadenotucirev DNA in tumors

Viral genomic DNA was detected in three in five fresh frozen biopsies ~5 weeks post-treatment (data not shown).

#### Viral kinetics and antibody response

For patients dosed with enadenotucirev IP, the virus was rarely detected in the blood at levels above the lower limit of quantitation of the assay. Viral shedding in buccal and rectal swabs or urine samples following IP dosing was only detected at levels below the lower limit of quantitation of the assay (data not shown). Among patients who received intravenous enadenotucirev, viral shedding was only detected at levels below the lower limit of quantitation of the assay (data not shown). Viral kinetics in the blood were as previously described for enadenotucirev.^10^ For patients who received intravenous enadenotucirev, an increase in anti-enadenotucirev antibody titer was seen (cycle 2, day 1 (day 29)) consistent with prior analyses^10^; similar increases were seen following IP dosing. The neutralizing potential of these antibodies was not tested.

## Discussion

Based on preclinical data demonstrating the activity of enadenotucirev in a model of platinum-resistant ovarian cancer and in vivo results showing potential synergy between enadenotucirev and paclitaxel,^12^ a phase 1 dose-escalation and dose-expansion study was conducted to assess the MTD and activity of enadenotucirev plus paclitaxel in platinum-resistant metastatic ovarian cancer. The combination of enadenotucirev 1×10^12^ vp administered intravenously and paclitaxel in patients with heavily pretreated ovarian cancer had a manageable tolerability profile and was associated with preliminary signals of efficacy.

As the use of IP paclitaxel and cisplatin chemotherapy had previously been shown to be associated with significantly improved PFS in advanced ovarian cancer,^4^ the OCTAVE study was initially designed to assess enadenotucirev given via an IP catheter. However, the incidence of catheter site complications, difficulties with this route of administration and the slow recruitment of patients into this cohort precluded determination of an an MTD of enadenotucirev given IP. Catheter complications and treatment discontinuation have also since been reported in other studies of IP dosing in ovarian cancer.^18^ This experience demonstrated that IP delivery of any therapy in this population is very challenging and probably should not be further attempted. Therefore, the route of administration of enadenotucirev was subsequently changed to intravenous and the starting dose of 1×10^12^ vp was used for the dose-expansion phase.

The safety profile observed here was consistent with previous data with enadenotucirev and paclitaxel alone,^10 17 19^ with no new safety findings and no evidence of enadenotucirev-related hepatic or renal injury. Five SAEs considered possibly related to enadenotucirev occurred; however, the overall safety profile observed suggests higher doses of intravenous enadenotucirev in combination with paclitaxel could have been investigated. Doses beyond 1×10^12^ vp were not assessed in combination with paclitaxel based on data from the EVOLVE study which determined an MTD of 3×10^12^ vp for intravenous enadenotucirev monotherapy, largely due to the occurrence of acute respiratory toxicities.^10^ No DLTs or cases of hypoxia were observed in OCTAVE, and subsequent studies have shown that higher doses of enadenotucirev can be tolerated with modifications to the dosing strategy. For example, a ‘low-high-high’ dose of intravenous enadenotucirev using a slower infusion rate and a lower first dose of 1×10^12^ vp on day 1, followed by up to 6×10^12^ vp on days 3 and 5 has now been assessed and shown to be tolerable in the SPICE study.^20^

Median PFS with intravenous enadenotucirev plus paclitaxel (6.2 months) was comparable to that observed with weekly paclitaxel in the SaPPrOC (5.3 months), OCTOPUS (4.2 months), and CARTAXHY studies (3.7 months).^17 19 21^ Although encouraging, this finding should be interpreted with some caution given the low number of patients assessed, which limits the precision of this estimate. Median PFS by Investigator assessment was lower at 3.7 months. The difference in the PFS estimates between Investigator and Independent assessments was likely due to a higher rate of censoring in the Independent assessments.

Response rates per Independent assessment appeared to be lower than those previously reported with weekly paclitaxel in similar populations^17 19 21^; however, stable reductions in target lesion burden were observed for up to 60 weeks and response rates per Investigator were more comparable to previous studies. Both response rate and the proportion of patients with a best overall response of PD was higher in Investigator vs Independent assessments, while more patients were considered to have a non-evaluable best overall response in Independent (n=6) vs Investigator assessments (n=1). Notably, all patients in this population had received prior paclitaxel, and approximately three-quarters of patients had received multiple prior lines of therapy containing paclitaxel, so the observation of antitumor activity with enadenotucirev plus paclitaxel in this study is encouraging.

OS data at the database lock remained immature for intravenous enadenotucirev plus paclitaxel (median not reached); an additional ad hoc follow-up demonstrated a median OS of 14.1 months. This OS value is promising when compared with previously reported values for patients receiving weekly paclitaxel for platinum-resistant ovarian cancer, for example, a median OS of 12.3 months was reported in the SaPPrOC study.^17^ However, as with the PFS results, this finding should also be interpreted with caution given the low number of patients. Similar data showing encouraging OS values, despite a low response rate, have been observed with enadenotucirev when given in combination with the PD-1 inhibitor nivolumab in the SPICE study in colorectal cancer.^22^

In five of six patients with paired biopsies, intratumoral CD8+ T-cell infiltration was observed approximately 5 weeks after treatment suggesting a possible proinflammatory effect driven by delivery and replication of enadenotucirev in these tumors. Only a small number of patients had sufficient tumor samples for analysis, and no clear correlation between T-cell infiltration and efficacy was observed. Of note, of the six patients assessed the patient with the highest number of post-treatment cytotoxic T cells also had a notable reduction in tumor burden; however, further assessment will be required to confirm any relationship between enadenotucirev, immune-cell infiltration and efficacy.

Viral genomic DNA was detected in three in five of available tumor samples ~5 weeks post-treatment demonstrating the persistence of enadenotucirev in tumors. The small size of the core biopsies and the heterogenous nature of viral infection in the tumor likely explains why the virus was not detected in all samples.

Overall these data show promising preliminary results for enadenotucirev plus paclitaxel in this heavily pretreated population, and suggest that intravenous administration of a tumor-selective adenovirus is feasible in combination with chemotherapy for platinum-resistant ovarian cancer. The use of next-generation viral vectors encoding and delivering immune-activating therapies within the tumor microenvironment may improve the efficacy of adenovirus-based therapy, and also help to further ameliorate highly immunosuppressive tumor microenvironments to improve the activity of immune-checkpoint inhibitors in a number of cancers, including ovarian cancer.^23 24^ Next-generation armed variants (Tumor-Specific Immuno Gene Therapy vectors) of enadenotucirev^25^ designed to reprogram the tumor microenvironment by expressing immune-enhancer transgenes (eg, cluster of differentiation (CD)−40 antibody or fibroblast activation protein and human CD3ε bispecific T cell activator antibodies) have demonstrated encouraging translational data^26 27^ and clinical trials are ongoing (NCT04830592, NCT04053283, NCT03852511).^28–30^

## Conclusions

The results of this study indicate that enadenotucirev can be administered intravenously, but not IP, in combination with paclitaxel with manageable tolerability and no DLTs in patients with heavily pretreated ovarian cancer. The safety information gathered in this study, taken together with the increased immune-cell infiltration, encouraging PFS and signs of durable responses suggest that further exploration of tumor-selective viruses in ovarian cancer is warranted.

## Data Availability

Data are available on reasonable request. The datasets generated and/or analyzed during the current study are not publicly available, however, any reasonable requests for access to available data underlying the results reported in this article will be considered. Such proposals should be submitted to the corresponding author.
