# Supplementary material for: Safety and efficacy of the tumor-selective adenovirus enadenotucirev with or without paclitaxel in platinum-resistant ovarian cancer: a phase 1 clinical trial
Source: J Immunother Cancer. 2021 Dec 10;9(12):e003645. doi: 10.1136/jitc-2021-003645 (PMC8666888; doi:10.1136/jitc-2021-003645)
Supplement: Supplementary data [file jitc-2021-003645supp001.pdf]

SUPPLEMENTARY APPENDIX

METHODS

Histopathology and immunohistochemistry

Immunohistochemical staining was performed on 4 µm thick sections of formalin-fixed paraffin embedded (FFPE) tumour tissue from pre- and post-treatment patient biopsies. Sections were baked for 2h at 60ºC and stained using duplex chromogenic IHC assays for pan-cytokeratin (PanCK)/CD8, CD8/Granzyme B or CD8/Ki67 on the Ventana Discovery Ultra platform at Histogenex (Wilrijk, Belgium). Additional tissue sections were stained for CD4, PD-1 and PD-L1. Antibodies used were CD4 (clone SP35, Roche), PD-1 (clone SP269, Abcam), PD-L1 (PD-L1 IHC 28-8 pharmDx kit, Agilent), CD8 (clone SP239, Abcam [CD8/PanCK assays]; clone C8/144B, Agilent [CD8/Ki67 assays]), Granzyme B (clone EPR8260, Abcam), PanCK (clone AE1/AE3/PCK26, Ventana) and Ki67 (Clone 30-9, Roche). CD8 positive cells in tumour epithelium and stroma and intratumoural CD8/granzyme B positive cells were detected using automated image analysis.

Gene expression profiling

Gene expression analysis was conducted on FFPE tumour tissue from pre- and post-treatment patient biopsies using the nCounter Analysis System (Nanostring Technologies) with the PanCancer Immune Profiling Panel codeset and a custom-designed codeset of 30 genes. Each hybridization reaction contained 50 ng of RNA, where RNA concentration permitted. Samples tested with a mean housekeeping gene RNA count of <100 were omitted from analysis as outliers. Genes that were expressed below calculated background levels were identified in each sample, and data were normalized using the NanoStringNorm package (version 1.2.1) in R (version 3.6.1).

Detection of enadenotucirev in tumour samples by qPCR

Detection of enadenotucirev in frozen tumour tissue from pre- and post-treatment patient biopsies was performed by qPCR of the E3 gene. DNA was extracted using the DNeasy

Blood & Tissue Kit (Qiagen). DNA was amplified using the following primers/probe; forward- ATC CAT GTC TAG ACT TCG ACC CAG, reverse- TGC TGG GTG ATA ACT ATG GGG T, probe- [FAM] ATC TGT GGA GTT CAT CGC CTC TCT TAC G [TAMRA], using Taqman™ Fast Advance Master Mix (Thermo Fisher Scientific). Each sample was tested in triplicate and the mean for each sample calculated.

RESULTS

Supplementary Table 1. PFS by Independent assessment (CGIG CA-125 and immune-related response criteria) and Investigator assessments (RECIST v1.1)

	Enadenotucirev IP Monotherapy (N=10)	Enadenotucirev IP + Paclitaxel (N=8)	Enadenotucirev IV + Paclitaxel (N=20)
**Independent Assessment**			
**GCIG CA-125, % (95% CI)**			
Median	3.9 (3.5, 4.4)	NE (3.3, NE)	6.1 (2.8, NE)
4-month PFS rate	50.0 (0.6, 91.0)	50.0 (0.6, 91.0)	72.2 (35.3, 90.3)
6-month PFS rate	0.0 (NE, NE)	0.0 (NE, NE)	72.2 (35.3, 90.3)
**irRC, % (95% CI)**			
Median	1.7 (1.2, 2.2)	3.3 (1.3, NE)	4.2 (2.0, 13.1)
4-month PFS rate	0.0 (NE, NE)	41.7 (1.1, 84.3)	56.5 (29.3, 76.7)
6-month PFS rate	0.0 (NE, NE)	0.0 (NE, NE)	32.3 (10.6, 56.6)
**Investigator Assessment**			
**RECIST v1.1**			
Median, % (95% CI)	1.7 (1.2, 2.2)	2.5 (1.3, 4.9)	3.7 (1.7, 5.4)
4-month PFS rate	15.9 (0.8, 49.6)	25.0 (1.2, 64.6)	42.1 (20.4, 62.5)
6-month PFS rate	0.0 (NE, NE)	0.0 (NE, NE)	21.1 (6.6, 41.0)
Min, Max	0.0, 5.2	0.0, 4.9	0.0, 9.5

RECIST=Response Evaluation Criteria in Solid Tumours; GCIG=Gynecological Cancer Intergroup; CA-125=Cancer Antigen 125; irRC=immune-related Response Criteria; CI=Confidence Interval; NE=Not estimable.

Supplementary Table 2. Overall survival in the full analysis set

	Enadenotucirev IP Monotherapy (N=10)	Enadenotucirev IP + Paclitaxel (N=8)	Enadenotucirev IV + Paclitaxel (N=20)
**Overall survival**			
Number of events/number censored (n)	**5/5**	**4/4**	**5/15**
Median, months	**8.0**	**6.4**	**NE**
95% CI	**3.5, 13.3**	**0.4, 16.5**	**6.8, NE**
Min, max	**1.6, 13.3**	**0.4, 16.5**	**0.4, 14.1**

CI=Confidence Interval; NE=Not estimable.

Supplementary Table 3. Response rate by Independent assessment (CGIG CA-125 and immune-related response criteria) and Investigator assessments (RECIST v1.1)

	Enadenotucirev IP Monotherapy (N=10)	Enadenotucirev IP + Paclitaxel (N=8)	Enadenotucirev IV + Paclitaxel (N=20)
**Independent Assessment**			
**Response Rate (GCIG CA-125)**			
Best Overall Response, n (%)			
Complete Response	0	0	2 (10)
Partial Response	0	0	2 (10)
No Response	1 (10)	0	0
Progressive Disease	0	0	2 (10)
Not Evaluable	9 (90)	8 (100)	14 (70)
Response Rate, %	0	0	20
95% CI	–	0.0, 36.9	5.7, 43.7
**irRC**			
ir Best Overall Response, n (%)			
ir Complete Response	0	0	0
ir Partial Response	0	0	2 (10)
ir Stable Disease	1 (10)	5 (63)	12 (60)
ir Progressive Disease	0	0	1 (5)
ir Not Evaluable	9 (90)	3 (38)	5 (25.0)
Response Rate, %	0	0	10
95% CI	–	0.0, 36.9	1.2, 31.7
Clinical Benefit Rate, %	10	63	70
95% CI	–	24.5, 91.5	45.7, 88.1
**Investigator Assessment**			
**RECIST v1.1**			
Best Overall Response, n (%)			

Complete Response	0	0	0
Partial Response	0	0	6 (30)
Stable disease	1 (10)	1 (13)	2 (10)
Progressive disease	6 (60)	4 (50)	11 (55)
Not Evaluable	3 (30)	3 (38)	1 (5)
Response rate, %	0	0	30
95% CI	–	0, 36.9	11.9, 54.3
Clinical benefit rate, %	10	13	40
95% CI	–	0.3, 52.7	19.1, 63.9

Supplementary Figure 1. Pre-clinical intraperitoneal dosing of enadenotucirev in combination with paclitaxel

A)

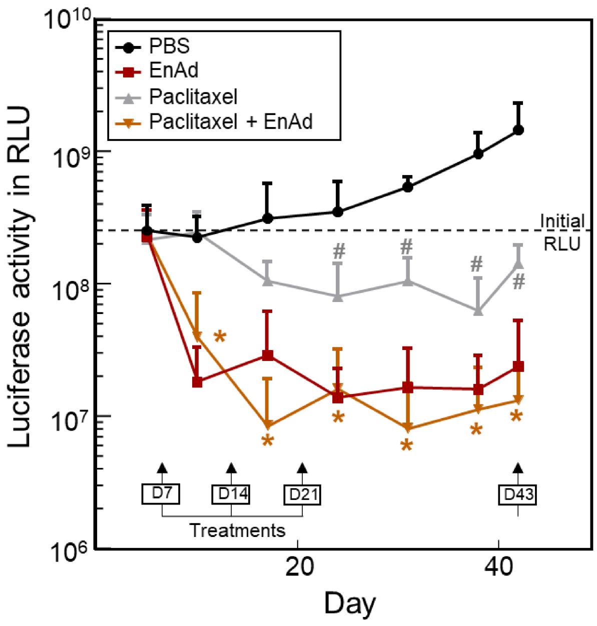

B)

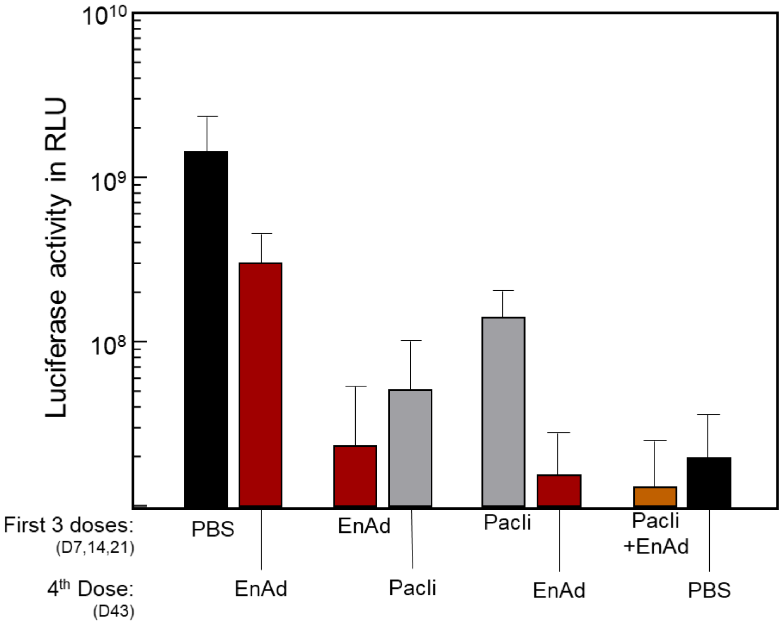

Female severe combined immunodeficient (SCID) mice (CB17) were implanted with luciferase-expressing SKOV-3 cells via intraperitoneal (IP) injection (2.5 x 10^6 cells per mouse in 2ml PBS) and subsequently dosed three times, 7 days apart, with paclitaxel, enadenotucirev, both combined or vehicle (PBS) via IP injection. All treatments were administered at a volume of 2 mL. Tumour burden was measured approximately weekly as *in situ* luciferase activity by *in vivo* imaging following luciferol administration and expressed as relative light units (RLU). At Day 43, mice were re-dosed with the agent not used previously, either enadenotucirev, paclitaxel or PBS, and tumor burden again measured three days later (D46). Enadenotucirev was administered at a dose of 5 × 10^9 viral particles and paclitaxel at a dose of 0.4 mg. A) Tumour burden over time, prior to treatment switch. B) Tumour burden at Days 42 (prior to treatment switch) and Day 46 (post-treatment switch). EnAd, enadenotucirev; Pacli, paclitaxel

# p<0.05 compared to PBS group; *P<0.05 compared to the PBS group and the paclitaxel only group.

Supplementary Figure 2.

Change in tumour burden over time (A) and best change in target lesion burden (B) per investigator assessment (RECIST 1.1; patients receiving IV enadenotucirev plus paclitaxel)

A)

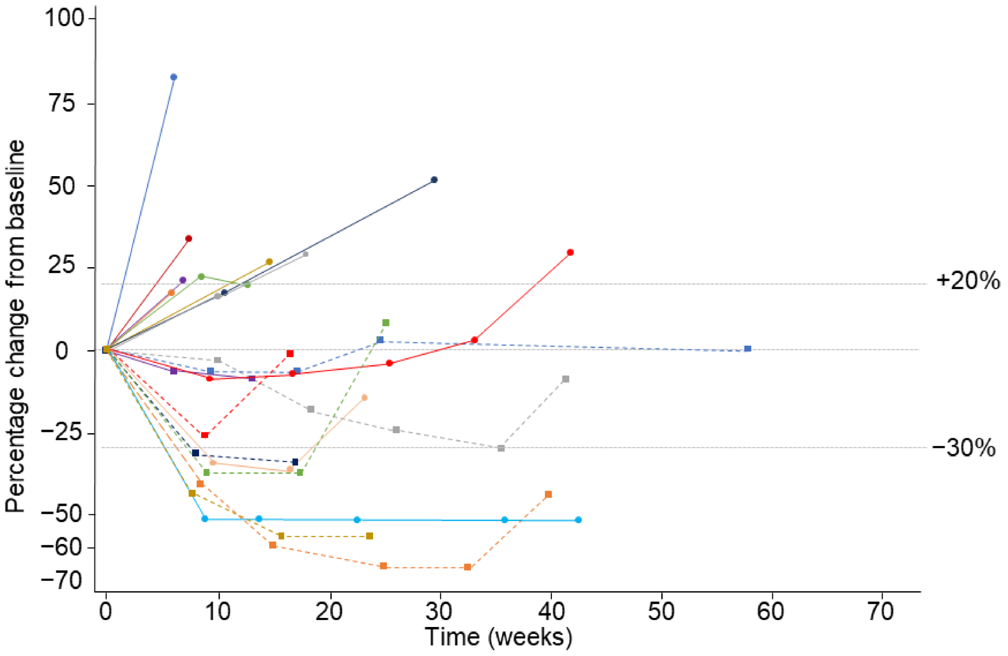

B)

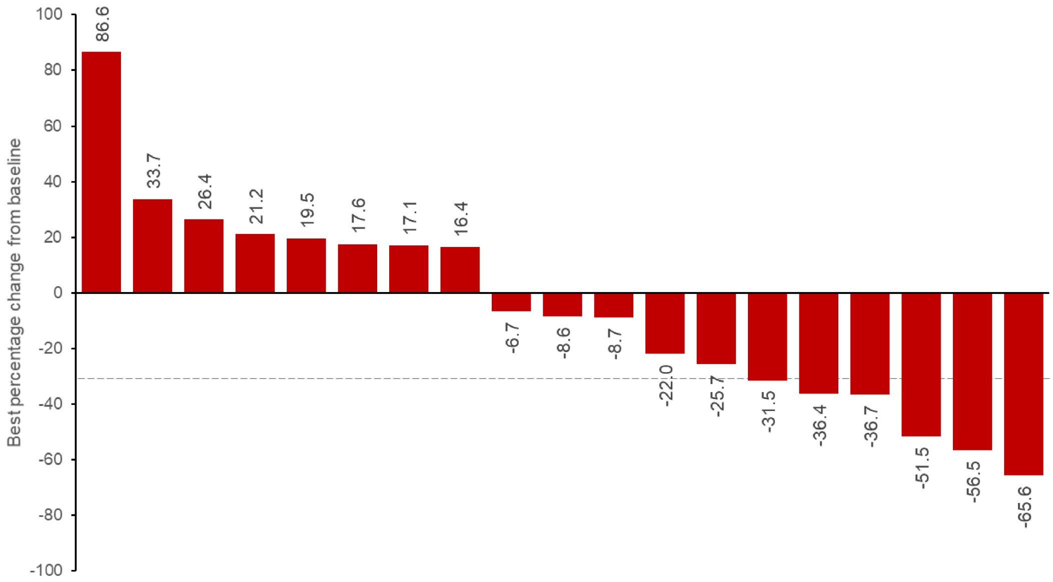

Evaluable patients: n=19. **A)** Percentage change from baseline in target lesion burden over time in the phase 1b enadenotucirev IV plus paclitaxel cohort. Each line represents an individual patient. Line colour for each individual patient is matched to that in Figure 3A. **B)** Best percentage change from baseline in target lesion burden (sum of diameters of target lesions per RECIST v1.1). Dashed line indicates 30% decrease in target lesion burden. Each bar represents an individual patient.
